# Identification of candidate long non-coding RNAs in response to myocardial infarction

**DOI:** 10.1186/1471-2164-15-460

**Published:** 2014-06-10

**Authors:** Jennifer Zangrando, Lu Zhang, Melanie Vausort, Fatiha Maskali, Pierre-Yves Marie, Daniel R Wagner, Yvan Devaux

**Affiliations:** Laboratory of Cardiovascular Research, Centre de Recherche Public de la Santé (CRP – Santé), 84 Val Fleuri, Luxembourg, L1526 Luxembourg; Nancyclotep Experimental Imaging Platform, Nancy, France; Division of Cardiology, Centre Hospitalier, Luxembourg, Luxembourg

**Keywords:** Myocardial infarction, Left ventricular remodeling, Non-coding RNAs

## Abstract

**Background:**

Long non-coding RNAs (lncRNAs) constitute a novel class of non-coding RNAs. LncRNAs regulate gene expression, thus having the possibility to modulate disease progression. In this study, we investigated the changes of lncRNAs expression in the heart after myocardial infarction (MI).

**Results:**

Adult male C57/BL6 mice were subjected to coronary ligation or sham operation. In a derivation group of 4 MI and 4 sham-operated mice sacrificed 24 hours after surgery, microarray analysis showed that MI was associated with up-regulation of 20 lncRNAs and down-regulation of 10 lncRNAs (fold-change >2). Among these, 2 lncRNAs, called myocardial infarction-associated transcript 1 (MIRT1) and 2 (MIRT2), showed robust up-regulation in the MI group: 5-fold and 13-fold, respectively. Up-regulation of these 2 lncRNAs after MI was confirmed by quantitative PCR in an independent validation group of 8 MI and 8 sham-operated mice (9-fold and 16-fold for MIRT1 and MIRT2, P < 0.001). In a time-course analysis involving 21 additional MI mice, the expression of both lncRNAs peaked 24 hours after MI and returned to baseline after 2 days. In situ hybridization revealed an up-regulation of MIRT1 expression in the left ventricle of MI mice. Expression of MIRT1 and MIRT2 correlated with the expression of multiple genes known to be involved in left ventricular remodeling. Mice with high level of expression of MIRT1 and MIRT2 had a preserved ejection fraction.

**Conclusion:**

Myocardial infarction induces important changes in the expression of lncRNAs in the heart. This study motivates further investigation of the role of lncRNAs in left ventricular remodeling.

**Electronic supplementary material:**

The online version of this article (doi: 10.1186/1471-2164-15-460) contains supplementary material, which is available to authorized users.

## Background

Cardiac diseases including stroke continue to be the main cause of death and disability in developed countries [1, 2]. Despite modern reperfusion strategies, a still significant proportion of patients develop left ventricular (LV) remodeling leading to heart failure after myocardial infarction (MI). Oxygen and nutrient deprivation to the heart induces severe damages, which can be of multiple types: necrosis or apoptosis of cardiac cells, cardiomyocyte hypertrophy, or fibrosis. Part of these damages can be induced by a de-regulation of gene expression.

Since the initial sequencing of the human genome more than a decade ago [3, 4], huge progress has been made in the understanding of its complexity. It appears now that only a minor part of the human DNA encodes proteins, while the remaining is transcribed into non-protein coding RNAs [5–7]. Non-coding RNAs have been arbitrarily dichotomized as short non-coding RNAs (20–22 nucleotides-long, called microRNAs, miRNAs) and long non-coding RNAs (lncRNAs, >200 nucleotides). While miRNAs down-regulate gene expression mostly by destabilization of target messenger RNA [8], the regulation of gene expression by lncRNAs appears to be much more complex, involving both activation and repression of gene expression, and modulation of chromatin architecture [9]. Since their discovery [10, 11], lncRNAs have emerged as attracting biomarkers and therapeutic targets in the oncology field. However, our knowledge of the role of lncRNAs in cardiovascular disease is only at its infancy.

Only few studies reported associations between lncRNAs and the heart. Two landmark studies reported the identification of 2 lncRNAs involved in cardiac development, Braveheart [12] and Fendrr [13]. In a genetic association study, the lncRNA MIAT (myocardial infarction-associated transcript) has been shown to be associated with the risk of MI [14]. Recent studies reported dysregulation of lncRNA expression in the failing heart [15, 16]. However, the role of lncRNAs in the infarcted heart is still poorly characterized. In particular, whether lncRNAs may affect the course of LV remodeling post MI is unknown.

The present study was designed to (1) determine the effect of MI on the expression of lncRNAs in the heart, and (2) identify lncRNAs potentially involved in LV remodeling post MI.

## Methods

### Animal experiments

This study was conducted in accordance with the regulations of the Animal Welfare Act of the National Institutes of Health Guide for the Care and Use of Laboratory Animals (NIH Publication No.85–23, revised 1996). Protocols were approved by the Regional Veterinary Department (‘Direction Départementale de la Protection des Populations’), agreements RAR1A03516811825 and 54–100.

Mice were anesthetized by inhalation of isoflurane/oxygen mixture (2.5%/1.5 v/v). When the mice were unresponsive to toe-pinch, they were intubated and ventilated with a rodent respirator and were placed on a heating pad. A left thoracotomy of the third interrib space was performed to expose the heart. After pericardial incision, permanent occlusion of the anterior interventricular artery was performed with a 7–0 Prolene suture. Having confirmed the presence of myocardial infarction by observation of ventricular blanching, ribs were closed with a 6.0 Vicryl suture, muscles were repositioned and the skin was sutured. The endotracheal tube was removed after spontaneous breathing. After surgery, mice were placed in an incubator at 30°C for at least 30 min and then returned to their cages.

For FDG-PET exam, mice received a pre-medication of 100 mg/kg of Acipimox in two intraperitoneal injections. The first dose was injected 1 hour before the injection of FDG and the second dose simultaneously to FDG injection. Pre-medication with Acipimox allows a higher myocardial uptake of FDG and enhanced signal to noise and myocardial to blood activity ratios [17]. One hour before the exam, 37 MBq of FDG was injected in tail vein. Recording was performed during 40 min under continuous isoflurane anaesthesia using a dedicated small animal PET system (Inveon, Siemens, Knoxville, TN, USA). FDG uptake was determined on collapsed short-axis slices in each segment from the 17-segment LV division from the American Heart Association [18] using the QPS software [19]. Left ventricular (LV) end-diastolic volume (EDV), LV end-systolic volume (ESV) and LV ejection fraction (EF) were obtained from contiguous ECG-triggered short-axis slices using the QGS software.

Mice were sacrificed with an overdose of isoflurane/oxygen mixture. Blood was harvested by cardiac puncture. Heart was excised and immediately homogenized in Lysis Binding Buffer (mirVana isolation kit, Life technologies) for extraction of total RNA using the mirVana isolation kit (Life technologies, Merelbeke, Belgium) according to manufacturer’s instructions. On-column DNase I digestion (Qiagen, Venlo, The Netherlands) was performed to eliminate potential contamination with genomic DNA. Concentration and integrity of RNA were assessed using a Nanodrop spectrophotometer (Nanodrop products, Wilmington, USA) and a 2100 Bioanalyzer (Agilent technologies, Santa Clara, USA), respectively.

### Microarrays

Agilent microarray platform was used with Low input Quick Amp labeling kit (Agilent) according to manufacturer’s instructions. Briefly, one-color spike mix was added to 200 ng of total RNA prior to amplification and labeling steps. Complementary RNA was purified with RNeasy Mini kit (Qiagen) and hybridized onto mouse SurePrint G3 microarrays (8x60K, Agilent). High-resolution microarray scanner (Agilent) and Feature Extraction software were used to scan the slide and extract raw microarrays data. Microarray data are available in the NCBI Gene Expression Omnibus [http://www.ncbi.nlm.nih.gov/geo] under the accession number GSE46395.

Pre-processing of raw data was performed using Limma [20] and VSN [21] packages rooted in the statistical computing environment R. Spots with a signal that was not significantly greater than the corresponding background – flag automatically established by Feature Extraction – were removed. Normalization between arrays was performed with Agilent spike-in probes. Principal component analysis was performed with R package ClassDiscovery [22]. After removing control probes and the probes that were detected in less than 4 samples per microarray slide, differentially expressed transcripts were determined using the *t*-test procedure within Significance Analysis of Microarrays version 3.09 which uses data permutations to estimate false discovery rate for multiple testing [23]. 100 permutations were used in our analyses. Heatmaps were created using Cluster 3.0 and TreeView [24]. Functional annotation and enrichment analysis of differentially expressed genes were performed with DAVID [25] (The Database for Annotation, Visualization and Integrated Discovery).

### LncRNA identification and construction of correlation networks

Microarrays were re-annotated for lncRNAs on a probe level. Briefly, microarray probes without accession prefix NM (i.e. protein-coding), according to the manufacturer, were aligned with lncRNAs from lncRNAdb [26], RefSeq [27] and Ensembl ncRNA [28] databases using BLAST + [29] rooted in the Perl environment. Only the probes that perfectly matched lncRNAs and did not match protein-coding RNAs (NM prefix transcripts from RefSeq) were considered as probes for lncRNAs.

Spearman’s rank correlation coefficient between selected lncRNAs and other remodeling-related transcripts from Gene database was obtained using the R package WGCNA [30]. This package computes the correlation coefficients and significance is determined by Student’s test. Correlations with a *P* value < 0.05 were visualized under the form of a network using CytoScape (PMID: 21149340).

### Real-time quantitative PCR

One μg of total RNA was reverse transcribed using Superscript II reverse transcriptase (Life technologies). Controls without reverse transcriptase were performed to ensure the absence of genomic DNA amplification during PCR. Real-time PCR was performed with IQ SYBR Green supermix in a CFX96 apparatus (Bio-rad, Nazareth, Belgium). PCR primers were designed using the Beacon Designer software (Premier Biosoft, USA) (Additional file 1: Table S1). PCR conditions were as follows: 3 min at 95°C, 30 s at 95°C, and 1 min annealing-extension (40-fold). Optimal annealing-extension temperature was determined for each primer pair. The specificity of the PCR reaction was confirmed by melting curve analysis. GAPDH was chosen as housekeeping gene for normalization. Expression levels were calculated by the relative quantification method (ΔΔCt) using the CFX Manager 2.1 software (Bio-Rad). Inter-run calibrator was used to normalize inter-run variations between separate real-time PCR runs.

### In situ hybridization

Hearts were fixed in formalin during 24 hours, and embedded in paraffin. 5-μm thick sections were performed. Expression of MIRT1 was assessed by in situ hybridization using the miRCURY LNA™ microRNA ISH Optimization Kit (Exiqon, Vedbaek, Denmark) according to the manufacturer's instructions. A scramble probe was used as negative control. Briefly, after deparaffinization with xylene and ethanol, sections were permeabilized with proteinase K (1 μg/mL). Then, sections were incubated with 40nM double-DIG LNA^TM^ MIRT1 probe (Exiqon) in hybridization solution (Sigma-Aldrich, Diegem, Belgium). Sections were washed and incubated with blocking solution (Roche, Howald, Luxembourg), and then with sheep anti-DIG antibodies coupled to alkaline phosphatase (Roche). Revelation was performed with NBT-BCIP solution (Roche) and the reaction was stopped with KTBT solution. Nuclei were stained with Nuclear Fast Red (Sigma-Aldrich).

### In situ hybridization coupled to immunostaining

To determine the cellular localization of MIRT1, in situ hybridization was performed as described above, with sheep anti-DIG antibodies coupled to fluorescein instead of alkaline phosphatase. Then, slides were subjected to immunohistochemical staining with a rabbit polyclonal antibody against sarcomeric alpha-actinin (Abcam, Cambridge, UK) to detect cardiomyocytes, a rabbit monoclonal antibody against vimentin (Abcam) to detect fibroblasts, or a rat monoclonal antibody against CD45 (SantaCruz, Heidelberg, Germany) to detect leukocytes. Alexa Fluor® 635-coupled goat anti-rabbit antibody and Alexa Fluor® 633-coupled goat anti-rat antibody were used as secondary antibodies (Invitrogen, Merelbeke, Belgium). Vectashield was used to reveal nuclei. Images were recorded on a confocal microscope (Zeiss Laser Scanning Microscope LSM 510 Carl Zeiss Microscopy, Oberkochen, Germany) using the LSM 510 META software (Carl Zeiss Microscopy, Oberkochen, Germany).

### Statistical analyses

Results are presented as mean ± standard deviation (SD). Statistical analyses were performed with the SigmaPlot v11.0 software. The Shapiro-Wilk normality test preceded all analyses. t-test and Mann–Whitney test were used to compare two groups of continuous variables following Gaussian and non-Gaussian distributions, respectively. Correlations between 2 variables were assessed using the Spearman test. Multiple group comparisons were performed using one-way analysis of variance and pairwise comparisons were performed using the Holm-Sidak method. All tests were two-tailed. A *P* value <0.05 was considered significant.

## Results

### Induction of MI in mice − derivation group

**Figure 1:**
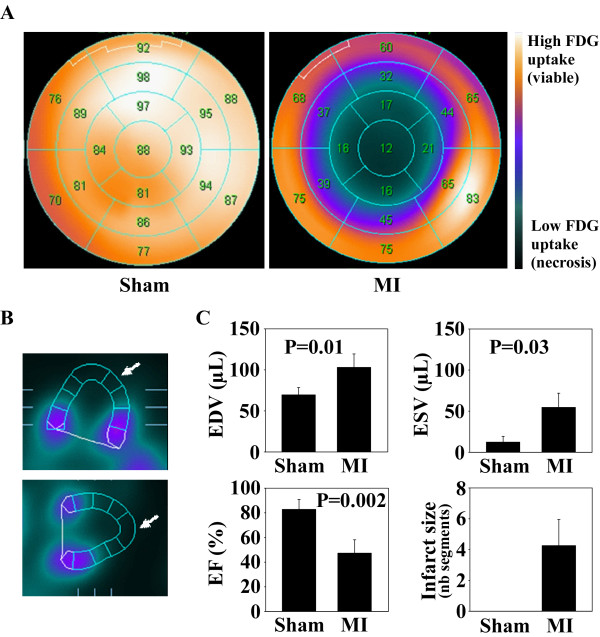
**FDG-PET exam in the derivation group.** Cardiac function was assessed in sham-operated mice (n = 4) and MI mice (n = 4) using FDG-PET, 24 hours after surgery. **A**. Representative polar maps of a sham-operated mouse and a MI mouse, in which the left ventricle is divided in 17 segments. In each segment, the indicated number corresponds to the percentage of FDG uptake. The segment was considered necrotic when the FDG uptake was inferior to 50%. Color scale shows the amount of FDG uptake, with darker color indicating lower FDG uptake (necrotic areas) and brighter color indicating high FDG uptake (viable areas). **B**. Vertical (up) and horizontal (bottom) long axis end-systolic PET images of a MI mouse. White arrows indicate necrotic areas (absent or low FDG uptake). **C**. LV volumes, EF and infarct size as determined by FDG-PET image analysis. Infarct size was evaluated by the number of necrotic segments (<50% FDG uptake).

A derivation group of 8 mice, 4 subjected to MI through coronary ligation and 4 subjected to sham-operation, was used to study the effects of MI on the expression of lncRNAs in the heart using microarrays.Left ventricular function was assessed in all mice by FDG-PET 24 hours after surgery. As shown in Figure 1, this exam allowed the visualization of infarcted areas of the heart in MI mice, which were absent in sham-operated mice. Considering that infarcted areas display less than 50% FDG uptake (due to loss of metabolic activity of the cells, and to cell death), we calculated infarct size for each mice, which is the number of infarcted segments related to the total number of 17 segments (see Methods for details). MI mice had in average 4 ± 2 infarcted segments, which correspond to a mean infarct size of 24% of the left ventricle. Sham-operated mice did not display any segment with less than 50% FDG uptake. LV end-diastolic and end-systolic volumes were significantly elevated, and the EF was reduced by 36% in average in MI mice compared to sham-operated mice.

### Microarray experiments − derivation group

**Figure 2:**
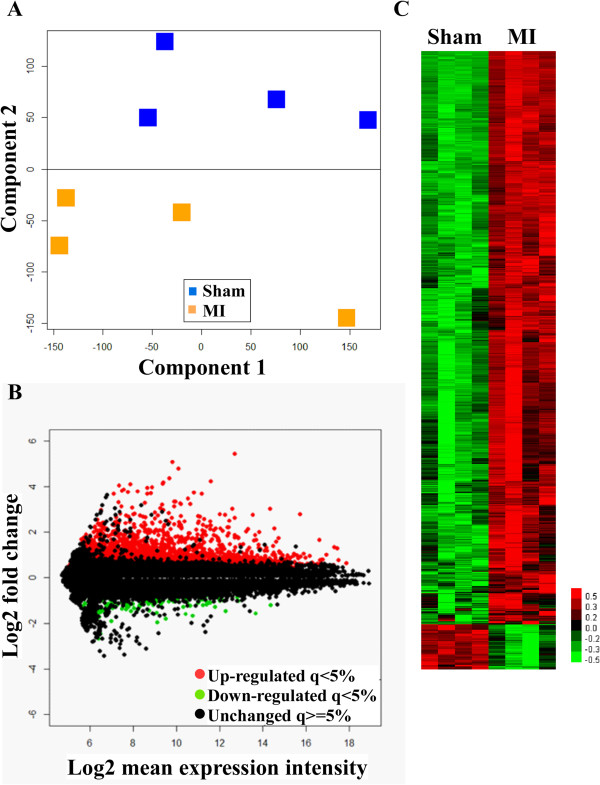
**Microarray analysis.** Cardiac transcriptome of 4 sham-operated mice and 4 MI mice (derivation group) was characterized 24 hours after surgery using microarrays. **A**. Principal component analysis showing the ability of gene expression data to discriminate MI mice from sham-operated mice. **B**. M-A plot showing the distribution of the genes in the dataset. The vertical axis displays log2 transformed-fold change and the horizontal axis displays the average signal of each gene. **C**. Heatmap of differentially expressed genes. For B and C, red color indicates genes up-regulated in MI mice and green color indicates genes down-regulated in MI mice compared to sham mice. Black color indicates genes with comparable expression between MI and sham mice. Significance threshold was 2-fold with a q-value <5%.

Total RNA from cardiac tissue of the 8 mice of the derivation group was extracted and used in microarray experiments. The Agilent SurePrint G3 Mouse Gene Expression microarray containing 55681 probes recognizing both coding and non-coding RNAs was used in these experiments. Microarray data were analyzed as described in Additional file 1: Figure S1. After pre-processing microarray data with R packages Limma and vsn, and after removing control probes and the probes that were detected in less than 4 of 8 microarrays, 34049 probes were retained for further analysis. Principal component analysis of these 34049 probes showed a clear discrimination of sham-operated and MI mice, demonstrating that MI significantly affected the cardiac transcriptome (Figure 2A). Principal component 1 accounted for 42% of total variability and principal component 2 accounted for 22% of total variability. Then, genes differentially expressed between sham-operated and MI mice were identified using significance analysis of microarrays method. Genes with a fold-change in expression >2-fold and a q-value <5% were considered as differentially expressed. 704 genes (918 probes) satisfied these criteria (Figure 2B-C).

Data mining using the DAVID database revealed that differentially expressed genes were highly involved in inflammation-related pathways (such as Cytokine-cytokine receptor interaction pathway, Chemokine signaling pathway, and Toll-like receptor signaling pathway) (Additional file 1: Table S2).

**Figure 3:**
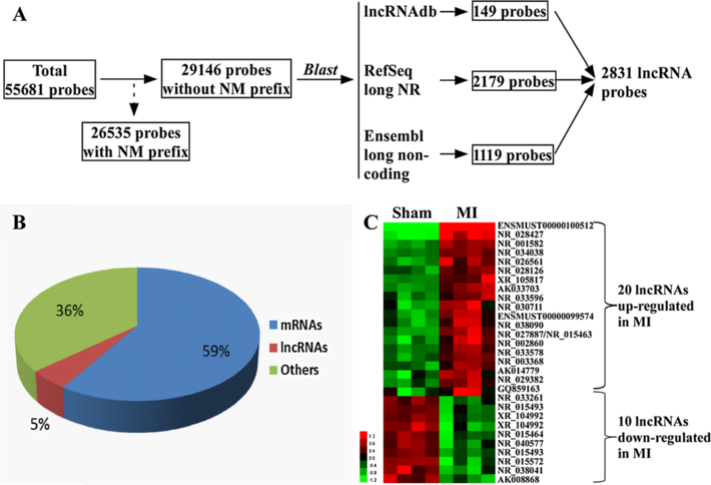
**Effect of MI on lncRNAs expression in the heart.** Microarrays performed with the 4 MI and 4 sham-operated mice (Derivation group) were mined for lncRNAs data. **A**. Analytical pipeline used to identify microarray probes recognizing lncRNAs. **B**. Percentage of probes on the microarray corresponding to mRNA and lncRNA transcripts. **C**. Heat-map of lncRNAs differentially expressed between MI and sham mice with a threshold fold-change of 2-fold and a q-value <5%. Red color indicates lncRNAs up-regulated in MI mice and green color indicates lncRNAs down-regulated in MI mice compared to sham mice.

We developed an analytical pipeline to extract lncRNA data from microarrays (Figure 3A). From the 55681 probes contained in the microarrays, we identified 26535 (48%) probes corresponding to transcripts with NM prefix (i.e. mature messenger RNAs, mRNAs). From the remaining 29146 probes, we identified 2831 probes corresponding to lncRNAs represented in lncRNAdb, RefSeq or Ensembl ncRNA database. These 2831 probes correspond to 5% of all probes on the microarray (Figure 3B). From the 2831 probes, significance analysis of microarrays identified 20 lncRNAs significantly up-regulated in the MI group and 10 lncRNAs down-regulated in the MI group (Figure 3C and Table 1). Therefore, this discovery phase allowed the identification of 30 lncRNAs regulated after MI.

**Table 1:** **LncRNAs differentially expressed between MI (n = 4) and sham-operated (n = 4) mice as determined by microarrays using a fold-change >2 and a q-value <5**%

Agilent systematic name	Agilent gene symbol	Identified lncRNA	Fold change	q-value(%)
ENSMUST00000100512	Gm10872	NR_045747 (Gm10872)	13.54	0.00
NR_028427	5830416P10Rik	NR_028427 (5830416P10Rik)	5.24	0.00
NR_002860	A130040M12Rik	VL30	2.71	0.00
chr2:158169020-158191295_R		NR_027887|NR_015463 (9430008C03Rik)	2.64	0.05
NR_001582	Speer5-ps1	NR_027506|NR_001582 (Speer5-ps1)	2.63	0.00
NR_026561	Gm8884	NR_026561 (Gm8884)	2.56	0.00
NR_030711	2210403K04Rik	NR_030711 (Mir22hg)	2.49	0.26
AK033703	Hmga2-ps1	NR_037996 (Hmga2-ps1)	2.48	0.00
XR_105817	3300005D01Rik	NR_045080|NR_045081|NR_045079 (3300005D01Rik)	2.34	0.00
chr5:23195569-23218551_F		NR_038090 (AI506816)	2.33	0.16
ENSMUST00000099574	Gm10791	NR_045889 (Gm10791)	2.31	0.00
AK014779	4833427F10Rik	NR_045459 (4833427F10Rik)	2.30	0.26
chr5:100849748-100858532_R		Adapt33 (NR_034038.1 Mouse 5430416N02Rik)	2.20	0.00
chr19:5771425-5848475_F		NEAT1	2.23	3.43
chr14:115443612-115445950_F		NR_029382 (Mir17hg)	2.16	0.16
NR_033578	Gm15645	NR_033578 (Gm15645)	2.13	0.00
NR_033596	5730416F02Rik	NR_033596 (5730416F02Rik)	2.11	0.05
chr5:74487385-74497428_F		NR_015531 (2700023E23Rik)	2.09	0.00
NR_003368	Pvt1	NR_003368 (Pvt1)	2.05	0.05
NR_028126	6330407A03Rik	NR_028126 (6330407A03Rik)	2.01	0.00
ENSMUST00000161216		ENSMUST00000161216 (2310075C17Rik)	0.36	1.82
NR_015572	1810014B01Rik	NR_015572 (1810014B01Rik)	0.38	1.82
chr10:69669025-69686275_F		NR_038041 (2310015B20Rik)	0.39	1.82
AK008868	2210409D07Rik	NR_045360 (2210409D07Rik)	0.42	1.82
chr4:11899775-11931875_F		NR_040577 (1700123M08Rik)	0.46	1.82
NR_015464	A330069E16Rik	NR_015464 (A330069E16Rik)	0.46	1.82
XR_104992	2310040G24Rik	NR_040292|NR_040293 (2310040G24Rik)	0.47	1.82
chr11:16831925-16851121_R		ENSMUST00000130392|ENSMUST00000139493| ENSMUST00000123734|ENSMUST00000136053| ENSMUST00000129735 (2810442I21Rik)	0.49	0.48
NR_033261	Gm14492	NR_033261 (Gm14492)	0.49	3.43
chrX:106029816-106082235_F		NR_015493 (2810403D21Rik)	0.50	3.43

### Validation of the effect of MI on lncRNAs

We sought to confirm by quantitative PCR the differential expression of the top 10 lncRNAs identified in microarray experiments in the derivation group (5 up-regulated and 5 down-regulated in MI mice compared to sham mice).

**Figure 4:**
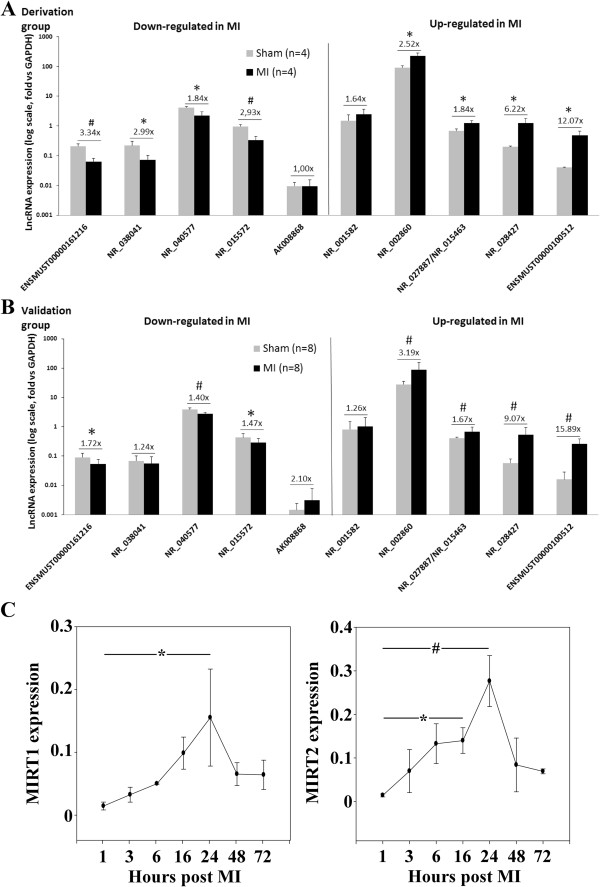
**Quantitative assessment of lncRNAs in the heart.** Expression of the top 10 lncRNAs identified as differentially expressed between MI and sham-operated mice in microarray experiments was quantified using quantitative RT-PCR, first **(A)** in the derivation group of 8 mice (4 sham and 4 MI), and then **(B)** in an independent validation group of 16 mice (8 sham and 8 MI). LncRNAs expression is shown relative to GAPDH (log scale). *P < 0.05; #P < 0.001 vs. sham-operated mice. **(C)** Time-course analysis of MIRT1 and MIRT2 in 21 additional mice subjected to coronary ligation and sacrificed after 1, 3, 6, 16, 24, 48, and 72 hours (n = 3 per time-point). *P < 0.05; #P < 0.001 vs 1 h time-point.

First, we used the RNA samples of the 8 mice enrolled in microarray experiments (Derivation group). We observed that some lncRNAs, such as NR_002860, were expressed at a high level, whereas others, such as AK008868, were expressed at a low level (Figure 4A). Differential expression was confirmed for 8 of the top 10 lncRNAs. Differential expression was not confirmed for AK008868, presumably due to its low level of expression and therefore low accuracy of its quantification by PCR. For NR_001582, the differential expression was present albeit not statistically significant. Noteworthy, 2 lncRNAs displayed robust up-regulation in the MI group: NR_028427 (6-fold, P = 0.03) and ENSMUST00000100512 (12-fold, P = 0.03). This is consistent with microarray results in which these 2 lncRNAs were also the most highly up-regulated in MI mice (5-fold and 13-fold for NR_028427 and ENSMUST00000100512, respectively; Table 1). We named NR_028427 as Myocardial Infarction-Associated Transcript 1 (MIRT1) and ENSMUST00000100512 as MIRT2.Second, we measured the expression levels of the same top 10 lncRNAs in an independent validation group of 16 mice (8 sham and 8 MI). The results obtained were very similar to that of the derivation group (Figure 4B). Indeed, differential expression was confirmed for 7 of the 10 lncRNAs. Statistical significance was lost for NR_038041, although the trend remained the same (slight down-regulation in the MI group). Most importantly, MIRT1 and MIRT2 were, like in the derivation group, the most up-regulated lncRNAs in MI mice, with increases of 9-fold (P < 0.001) and 16-fold (P < 0.001), respectively.Third, 21 additional mice were enrolled in a time-course analysis. Mice were subjected to coronary ligation and sacrificed after 1, 3, 6, 16, 24, 48, and 72 hours (n = 3 per time-point). Hearts were harvested and expression of MIRT1 and MIRT2 was assessed by quantitative PCR (Figure 4C). Expression of MIRT1 and MIRT2 displayed progressive increases after induction of MI, reaching a maximum of 10-fold and 19-fold after 24 hours.

Collectively, these results show that MI induces significant changes in lncRNAs expression in the heart.

### Localisation of MIRT1 in the heart

**Figure 5:**
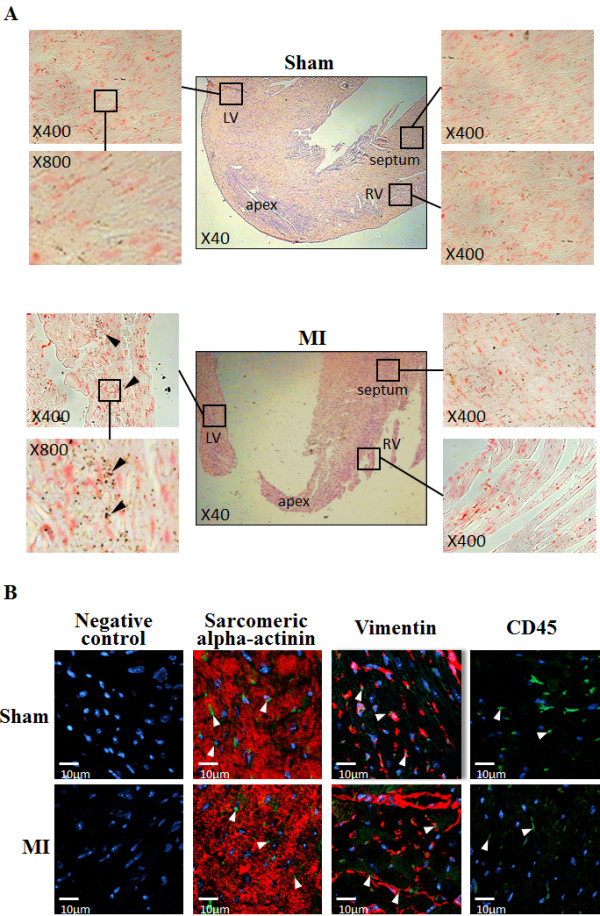
**Localisation of MIRT1 in the heart. (A)** In situ hybridization revealed that MIRT1 was present mostly in the remote area of the left ventricle, with higher expression observed in MI mice. Representative pictures from 3 sham and 3 MI mice are shown. Arrowheads point to areas with high expression of MIRT1. LV: left ventricle; RV: right ventricle. **(B)** In situ hybridization coupled to immunostaining revealed that MIRT1 was expressed by cardiac fibroblasts. Sarcomeric alpha-actinin was used to stain cardiomyocytes, vimentin was used for fibroblasts, and the pan-leukocyte antigen CD45 was used for leukocytes. MIRT1 staining appears in green color and is indicated by white arrow heads. Sarcomeric alpha-actinin, vimentin and CD45 appear in red color. Nuclei are blue. Negative control was performed by omission of primary antibody. Magnification: x400.

Three additional mice were subjected to coronary ligation and 3 were sham-operated. The presence of MIRT1 in the heart 24 hours after surgery was revealed using in situ hybridization. As displayed in Figure 5A, MIRT1 expression was up-regulated in the healthy part of the left ventricle of MI mice (remote area). In situ hybridization of MIRT1 coupled with immunostaining of cardiomyocytes (sarcomeric alpha-actinin), fibroblasts (vimentin) and leukocytes (CD45) revealed that MIRT1 was expressed by fibroblasts (Figure 5B).

### Correlation between lncRNAs, infarct size and LV function

We investigated the correlation between expression levels of the top 10 lncRNAs, infarct size and LV function as assessed by FDG-PET in the 4 MI mice of the derivation group, 24 hours after coronary ligation. There was no statistically significant correlation at the 5% level, presumably due to the low number of mice (Table 2). Nevertheless, relatively strong negative associations were observed between expression levels of MIRT1, MIRT2, and infarct size (correlation coefficients of −0.80). On the other hand, positive associations could be noticed between MIRT1, MIRT2 and EF (correlation coefficients of 0.85 and 0.80, respectively), suggesting that MIRT1 and MIRT2 may beneficially affect LV remodeling post MI.

**Table 2:** **Correlation between lncRNAs expression, infarct size and LV function as assessed by FDG-PET in 4 mice with MI of the derivation group**

	Infarct size	EDV	ESV	EF
**ENSMUST00000161216**	−0.20 (0.92)	0.38 (0.62)	0.20 (0.80)	−0.14 (0.86)
**NR_038041**	−0.80 (0.33)	−0.07 (0.93)	−0.57 (0.43)	0.75 (0.25)
**NR_040577**	−0.40 (0.75)	0.56 (0.44)	0.01 (0.99)	0.36 (0.64)
**NR_015572**	−0.20 (0.92)	0.10 (0.90)	−0.20 (0.80)	0.27 (0.73)
**AK008868**	0 (1)	−0.95 (0.05)	−0.69 (0.31)	0.29 (0.71)
**NR_001582**	0.40 (0.75)	−0.89 (0.11)	−0.59 (0.41)	0.17 (0.83)
**NR_002860**	−0.40 (0.75)	0.76 (0.24)	0.50 (0.50)	−0.10 (0.90)
**NR_027887/NR_015463**	−0.20 (0.92)	0.46 (0.54)	0.19 (0.81)	0.16 (0.84)
**NR_028427 (MIRT1)**	−0.80 (0.33)	−0.48 (0.52)	−0.75 (0.25)	0.85 (0.15)
**ENSMUST00000100512 (MIRT2)**	−0.80 (0.33)	0.60 (0.42)	0.00 (1.00)	0.80 (0.33)

Correlation coefficients (P value) are indicated.

### Association between MIRT1, MIRT2, and LV remodeling

**Figure 6:**
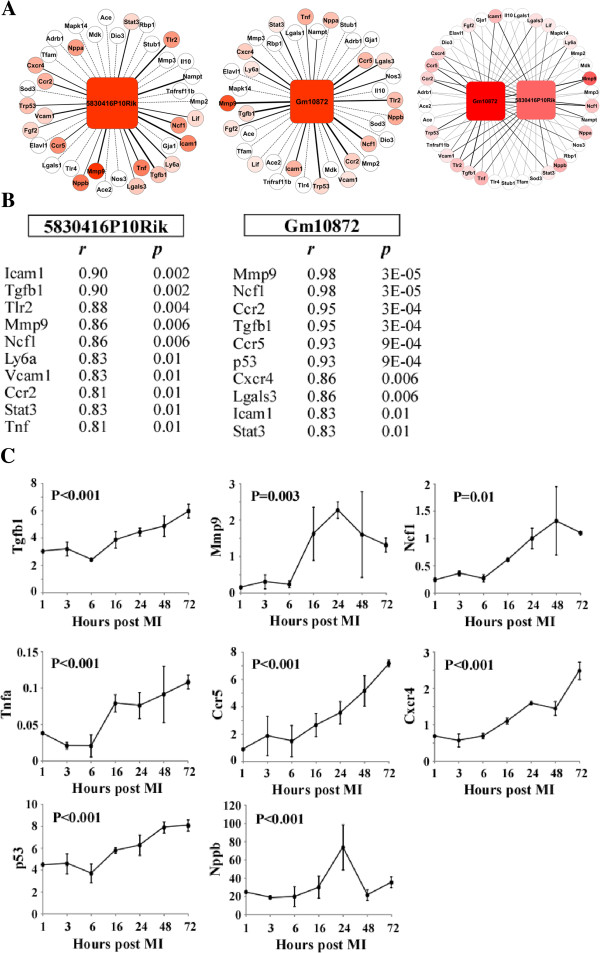
**Correlation between lncRNAs and remodeling genes.** Microarray data from the derivation group of 4 sham-operated and 4 MI mice were used in these analyses. **A**. Networks indicating the strength of the correlation between the lncRNAs MIRT1 and MIRT2 and 38 coding genes known to be involved in remodeling (“remodeling genes”). Remodeling genes differentially expressed between sham-operated and MI mice are coloured, with darker colour indicating a strong differential expression. Red colour indicates a higher level of expression in MI mice compared to sham-operated mice. Remodeling genes unaffected by MI are in white circles. A q-value <5% was used as threshold for differential expression between sham and MI mice (significance analysis of microarrays method). The thickness of the edges indicates the strength of the correlation between the lncRNAs and remodeling genes. Dotted lines indicate no correlation. A p-value <0.05 was used as significance threshold for correlation (Spearman’s rank correlation and Student’s test). **B**. List of the remodeling genes significantly (p < 0.05) correlated with lncRNAs and differentially expressed between sham-operated and MI mice. *r* indicates correlation coefficient and *p* indicates p-value. **C**. Kinetic of the expression of remodeling genes after MI. The 21 mice sacrificed at different time-points after MI were used in these analyses (n = 3 per time-point). P values obtained by ANOVA are indicated.

To gain further insight into the potential role of MIRT1 and MIRT2 into LV remodeling, we used the following approach. First, we identified a list of 47 genes known to be involved in remodeling using the keywords (“myocardial infarction” AND “ventricular remodeling”) AND (“homo sapiens”[Organism] OR “mus musculus”[Organism])” as a query to NCBI Gene database (=”remodeling genes”). Among these 47 remodeling genes, 38 genes were detected on microarrays. Then, we used the microarray data of the derivation group of 4 sham-operated and 4 MI mice to determine the correlation between the expression values of these 38 remodeling genes and the expression values of MIRT1 and MIRT2. This allowed building networks of correlation between the lncRNAs and the 38 remodeling genes (Figure 6A). MIRT1 was significantly correlated with 18 remodeling genes, and MIRT2 was significantly correlated with 17 remodeling genes. Interestingly, while both lncRNAs were correlated with the same remodeling genes, Lif was only correlated with MIRT1 and Nos3 was only correlated with MIRT2 (Figure 6A, right network). Figure 6B lists the 10 strongest correlations between lncRNAs and remodeling genes whose expression was regulated after MI. Correlations were highly robust, with coefficients close to 1 for some gene pairs. Finally, we characterized the regulation of the expression of some remodeling genes in the group of 21 mice sacrificed at different time-points post MI (Figure 6C). All genes were up-regulated. We also measured Nppb, which encodes BNP, and we observed that, while most remodeling genes remained elevated 3 days after MI, Nppb had returned to baseline levels after 48 hours. This kinetic was similar to that of MIRT1 and MIRT2.

Together, these data support an association between the lncRNAs MIRT1 and MIRT2, and genes known to be involved in LV remodeling.

## Discussion

In this study, we observed for the first time that MI induces a significant regulation of the expression of lncRNAs in the heart. Some of these lncRNAs were correlated with protein coding genes known to be involved in LV remodeling. These lncRNAs constitute novel candidates for future investigations of the therapeutic value of lncRNAs.

Four groups of mice were used in this study. First, a derivation group of 8 mice was used to profile the expression of lncRNAs using microarrays. FDG-PET exams scheduled 24 hours after induction of MI allowed to characterize infarct size and LV function. In average, infarct covered one fourth of the left ventricle. EF was reduced by 36% 24 hours after induction of MI, which attests for a significant loss of LV function. End-diastolic and end-systolic volumes were increased, consistently with LV dilatation. Second, a validation group of 8 independent mice was used to confirm microarray data. Third, a kinetic using 21 additional mice allowed characterizing the evolution of the expression of lncRNAs in the cardiac tissue after induction of MI. Fourth, 6 mice were used to study the localization of lncRNAs in the infarcted heart. This experimental design supports the robustness of our findings.

A whole genome microarray was used for the discovery phase of our study. We observed that MI affected the expression of a significant number of genes (704), allowing a clear discrimination of MI mice from sham-operated mice. Many of these genes had a known link with the regulation of inflammation. This was expected considering that mice were sacrificed in the early inflammatory phase that occurs in the first 24 hours post MI. Of note, this time-point was chosen to identify early triggers of LV remodeling which could be used to blunt or inhibit the development of LV remodeling at a very early stage after MI.

An in-house analytical pipeline was developed to extract lncRNAs data from microarrays. A similar approach has already been used elsewhere [31]. We could identify 30 lncRNAs whose expression was regulated more than 2-fold and with a q-value <5% following MI. Of note, 12 of these 30 lncRNAs were also dysregulated in the heart of isoproterenol-treated mice [15]. We then focused on the 2 lncRNAs most differentially expressed between MI mice and sham-operated mice, MIRT1 and MIRT2. Up-regulation of these lncRNAs after MI was consistently observed in all groups of mice, and peaked after 24 hours. This up-regulation might be, at least in part, attributed to infiltration of inflammatory cells into the heart. This is supported by microarray data showing an up-regulation of the leukocyte marker CD45 in MI mice compared to sham mice (2.1-fold) and of the monocyte/macrophage marker CD68 (1.6-fold). However, CD45-positive leukocytes could not be detected by immunostaining in the remote area of the heart where MIRT1 expression is observed. This suggested that the increase of CD45 and CD68 measured by microarrays come from inflammatory cells that are infiltrated in the infarct lesion. In situ hybridization coupled to immunohistochemistry confirmed that MIRT1 is mainly expressed by fibroblasts within the remote area of the left ventricle. Furthermore, expression levels of MIRT1 and MIRT2 appeared to be negatively correlated with infarct size and positively correlated with EF. Although this is consistent with the known impact of infarct size on LV function, large infarcts inducing a deterioration of LV function with decreased EF, it also strengthens our assumption that expression levels of MIRT1 and MIRT2 are not a mere consequence of inflammation.

Inflammation is an important component of the remodeling process. To address a potential link between the up-regulation of MIRT1 and MIRT2, and LV remodeling, we used microarray data to determine the correlations between the expression values of genes known to be involved in LV remodeling, and the expression values of MIRT1 and MIRT2. Strong correlations were observed, suggesting that these 2 lncRNAs may functionally regulate LV remodeling. However, this remains to be further explored and the contribution of these lncRNAs in the remodeling process further demonstrated.

It would be tempting to investigate the expression of MIRT1 and MIRT2 lncRNAs in the human failing heart. However, there are no known homologs of these lncRNAs in human.

In network analyses, both lncRNAs generally correlated with the same remodeling genes, except for Lif, which was only correlated with MIRT1 and Nos3, which was only correlated with MIRT2. This finding deserves further independent validation. The possible interaction between MIRT1 and Lif on one hand, and between MIRT2 and Nos3 on the other hand, as well as its potential role in LV remodeling, needs to be further addressed.

Nppb, the gene which encodes BNP, was correlated with MIRT1 and showed a 4-fold increase in expression after MI, peaking after 24 hours and returning to baseline levels after 48 hours. This kinetic was different from other tested remodeling genes, which all remained elevated until at least 72 hours after induction of MI. This observation merits to be validated but already points out a possible interaction between MIRT1 and BNP.

Lgals3, which encodes the lectin galactoside-binding soluble 3, more commonly known as galectin-3, was significantly correlated with MIRT1 and MIRT2. This observation is relevant and in line with the role of galectin-3 in fibrosis and with its recently characterized value as biomarker of heart failure [32, 33]. However, no evident relationship could be evidenced between circulating levels of galectin-3 and LV remodeling in survivors of acute MI [34].

Overall, our results suggest that lncRNAs may be involved in the regulation of several pathophysiological pathways leading to LV remodeling: inflammation (TNF), extracellular matrix turnover (MMP9), fibrosis (TGFB1 and LGALS3), apoptosis (p53).

## Conclusion

This hypothesis-generating study has led to the discovery of novel lncRNAs that may play functional roles in LV remodeling post MI. Further investigations are required to demonstrate the therapeutic potential of these lncRNAs.

### Availability of supporting data

The data set supporting the results of this article is available in the NCBI Gene Expression Omnibus repository [http://www.ncbi.nlm.nih.gov/geo] under the accession number GSE46395.

## Electronic supplementary material

Additional file 1: **Online supplement. Table S1.** Real-time quantitative PCR primers. **Table S2.** Mining of microarray data using the DAVID database. **Figure S1.** Detailed analytical pipeline for microarray experiments. (DOCX 57 KB)
