# Supplementary material for: Identification of candidate long non-coding RNAs in response to myocardial infarction
Source: BMC Genomics. 2014 Jun 10;15(1):460. doi: 10.1186/1471-2164-15-460 (PMC4070571; doi:10.1186/1471-2164-15-460)

**Additional file 1**

**ONLINE SUPPLEMENT**

**Table S1.** Real-time quantitative PCR primers.

| LncRNA | Accession # | Forward primer | Reverse primer | T °C |
| --- | --- | --- | --- | --- |
|  |  |  |  |  |
| 2310075C17Rik | ENSMUST00000161216 | ACAACAACAGATTAGATC | AGGACATTATAGCACTTA | 56 |
| 2310015B20Rik | NR_038041 | ACTGATCTCTACTACTTCG | TTGCTAGGACGTTATGAC | 56 |
| 1700123M08Rik | NR_040577 | ATGGCTACAGTTTGTGGA | TATTCACTCACAACAGCTT | 56 |
| 1810014B01Rik | NR_015572 | GCTGAGTTTAAGTATGTC | CTTTCCTTCATTTCTCTC | 56 |
| 2210409D07Rik | NR_045360 | CTCATCTACTAATCCCCAGAA | GCAAAACATTTTAATCACAACTG | 56 |
| Speer5-ps1 | NR_027506/  NR_001582 | AATCTTCCTTGGATGAATCTTCTT | CAGTCGCCTCAGTTCTCT | 54 |
| VL30 | NR_002860 | ACGTTCTCAAGATAGATAA | ACACACAATGGAATTTTC | 56 |
| 9430008C03Rik | NR_027887/  NR_015463 | ATCATCTAGTGCCTGGAA | ATGGGTTGACACAGATTT | 56 |
| 5830149P10Rik | NR_028427 | CTTGTTGTCTAAGTGAGTA | TTAATCGTTCCTCTAGTTG | 58 |
| Gm10872 | NR_045747 | TCAACACTTTCCATAGGT | ATTGTGAGGTCCAGATAG | 54 |
| Tgfb1 | NM_011577.1 | AGACATCTCACACAGTAT | CCAGGAATTGTTGCTATA | 60 |
| Mmp9 | NM_013599.3 | GCTGACTACGATAAGGAC | AAAGATGAACGGGAACAC | 52 |
| Ncf1 | NM_010876.3 | CAGGTGAACCGTATGTAA | CAGATACATGGATGGGAA | 56 |
| Tnf-alpha | NM_013693.1 | TCTTCTCATTCCTGCTTGTG | ACTTGGTGGTTTGCTACG | 56 |
| Ccr5 | NM_009917.5 | AACACCTGGATTATGAAT | CTGCTAAGTAAATTAGAGAA | 58 |
| Cxcr4 | NM_009911.3 | GCATCATCATCTCTAAGC | TAGGATGAGGATGACTGT | 56 |
| p53 | NM_011640.3 | GGAGTCACAGTCGGATAT | AAACTCCTCAACATCCTG | 60 |
| Nppb | NM_008726.4 | ATGATTCTGTTTCTGCTTT | ATTTCCTCCGACTTTTCT | 58 |

T °C: annealing temperature.

**Table S2.** Mining of microarray data using the DAVID database.

| **Term** | **Count** | **PValue** | **FDR (%)** | **Genes** |
| --- | --- | --- | --- | --- |
| Cytokine-cytokine receptor interaction | 46 | 5.68E-18 | 6.69E-15 | CXCL1, IL1R2, CCL3, TNF, CCL2, CXCL5, TNFRSF12A, CSF2RB2, CSF1, CXCL2, IL4RA, TNFSF14, CXCR2, KIT, CCL4, IL17RA, CCL7, TGFB2, IL11, CXCL10, CCL6, CLCF1, CXCR4, CXCR6, TNFRSF18, CSF2RB, IL1B, LTB, CSF2RA, IL6, BMP2, TNFRSF13B, TNFSF9, CCL17, INHBB, OSM, INHBA, CCL12, TNFRSF9, CCR7, CNTF, CCR5, RELT, PPBP, CCR2, CCR1L1 |
| Chemokine signaling pathway | 29 | 1.68E-09 | 1.98E-06 | CXCL1, CCL3, CCL2, FGR, CXCL5, NFKBIB, CXCL2, CXCR2, CCL4, CCL7, CCL6, CXCL10, RAC2, TIAM2, CXCR4, CXCR6, PIK3R5, PIK3CG, NCF1, HCK, VAV1, CCL17, CCL12, CCR7, ARRB2, PPBP, CCR5, CCR2, CCR1L1 |
| Hematopoietic cell lineage | 19 | 7.99E-09 | 9.41E-06 | IL1R2, IL6, TNF, CSF1, IL4RA, ITGA3, KIT, ITGB3, CD24A, ITGAM, IL11, CD37, TFRC, CD44, ITGA5, CD33, IL1B, CD14, CSF2RA |
| NOD-like receptor signaling pathway | 15 | 1.94E-07 | 2.29E-04 | CXCL1, IL6, TNF, CCL2, NFKBIB, CXCL2, CCL7, CCL12, NAIP2, MEFV, MAPK13, CASP8, PSTPIP1, IL1B, TNFAIP3 |
| Toll-like receptor signaling pathway | 18 | 6.37E-07 | 7.51E-04 | PIK3CG, CCL3, IL6, TNF, MAP2K3, TLR1, TLR2, TIRAP, TLR6, CCL4, CXCL10, IRF5, MAPK13, IRF7, CASP8, IL1B, PIK3R5, CD14 |
| Leukocyte transendothelial migration | 17 | 3.52E-05 | 4.15E-02 | PIK3CG, ICAM1, NCF2, NCF1, MMP9, NCF4, ACTN1, ITGB2, VAV1, VASP, ITGAM, ACTG1, RASSF5, RAC2, MAPK13, CXCR4, PIK3R5 |
| Regulation of actin cytoskeleton | 23 | 1.10E-04 | 1.30E-01 | GNA13, PIK3CG, ENAH, ARHGEF7, ACTN1, ITGB2, ITGA3, ITGB3, PIP5K1A, VAV1, ITGAM, PAK6, ACTG1, RAC2, TIAM2, ITGAX, ITGA5, PAK3, RRAS2, CFL1, PIK3R5, CD14, FGD3 |
| Fc gamma R-mediated phagocytosis | 14 | 2.21E-04 | 2.60E-01 | PIK3CG, PTPRC, MARCKSL1, NCF1, HCK, SPHK1, ARF6, PIP5K1A, VAV1, VASP, FCGR2B, RAC2, CFL1, PIK3R5 |
| Jak-STAT signaling pathway | 17 | 6.40E-04 | 7.51E-01 | PIK3CG, PTPN6, IL6, CSF2RB2, SOCS3, STAM2, IL4RA, SOCS1, PIM1, IL11, OSM, CNTF, CLCF1, CSF2RB, PIK3R5, MYC, CSF2RA |
| Natural killer cell mediated cytotoxicity | 14 | 1.83E-03 | 2.13E+00 | PIK3CG, ICAM1, PTPN6, TNF, FCGR4, ITGB2, VAV1, HCST, FCGR3, RAC2, FCER1G, PIK3R5, SH3BP2, TYROBP |
| Cytosolic DNA-sensing pathway | 9 | 2.00E-03 | 2.33E+00 | TMEM173, IL6, IRF7, NFKBIB, RIPK3, IL1B, CCL4, ZBP1, CXCL10 |
| Focal adhesion | 18 | 4.10E-03 | 4.72E+00 | PIK3CG, COL4A2, COL4A1, PGF, ACTN1, ITGA3, ITGB3, FLNC, FLNB, VAV1, VASP, ACTG1, PAK6, RAC2, PAK3, ITGA5, PIK3R5, THBS1 |

Pathways over-represented by differentially expressed transcripts with a false discovery rate (FDR) <5% are shown.

**Figure S1**. Detailed analytical pipeline for microarray experiments.


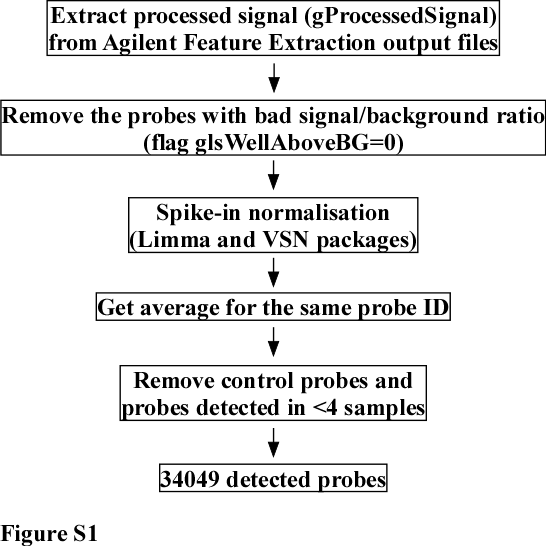

Supplement: Supplementary file 1 — Additional file 1: Online supplement. Table S1. Real-time quantitative PCR primers. Table S2. Mining of microarray data using the DAVID database. Figure S1. Detailed analytical pipeline for microarray experiments. (DOCX 57 KB) [file 12864_2014_6146_MOESM1_ESM.docx]
